# Editorial: Very early identification and intervention for infants with prodromes of autism

**DOI:** 10.3389/fpsyt.2026.1910179

**Published:** 2026-07-30

**Authors:** Costanza Colombi, Josephine Barbaro, Patrick Dwyer, So Hyun Kim

**Affiliations:** 1 Stella Maris Foundation (IRCCS), Calambrone, Italy; 2 La Trobe University, Melbourne, VIC, Australia; 3 Korea University, Seoul, Republic of Korea

**Keywords:** autism spectrum disorder, early development, early identification, infants, preemptive intervention, prodromal signs

Very early identification and intervention for infants with prodromes of autism demand a fundamental shift from “wait and see” toward “watch and do.” This Research Topic brings together work showing that early behavioral atypicalities are already measurable and clinically meaningful in the first year of life, while current systems remain poorly organized to act on them. Edwards et al. follow a large cohort of infants at multiple time points from 12- to 24-months, using the Communication and Symbolic Behavior Scales to map trajectories of social communication. They show that group differences present around the first birthday tend to persist or widen over time and are closely linked to 24-month receptive and expressive language outcomes, even when controlling for diagnosis and maternal education, underscoring early social communication as a strategic target for pre-emptive support.

Clintberg et al. add an important parental perspective by examining concerns reported by caregivers of infant siblings of Autistic children between 6- and 24-months. Infants diagnosed at 18-months had more concerns across domains than those diagnosed later, with particularly large differences in play, language, and language regression. Their study highlights that early autism prodromes are heterogeneous, that early absence of a diagnosis does not rule out a later one, and that systematic attention to parent concerns is itself a powerful early-identification strategy.

Motor development is placed at the center by Ting and colleagues’ systematic review of hypotonia during infancy. They show that early hypotonia and gross motor delay frequently precede core Autistic features’ emergence and are associated with later autistic traits and functional outcomes, even though they are not autism-specific, arguing that motor quality should be integrated into multidimensional developmental surveillance rather than treated as an incidental co-occurrence.

Dowds et al. present a pilot study of the Baby Social ABCs, an online caregiver-mediated intervention for infants as young as 6-months. Preliminary findings are encouraging: caregivers’ use of early support strategies increased, as did infant social communication behaviors, and caregivers found the program feasible and acceptable.

Conti et al. systematic review of randomized controlled trials of parent-mediated interventions in infants and toddlers with elevated likelihood for autism or very early signs (mean age below 18 months) offers an up-to-date synthesis. Across 11 trials representing seven cohorts, the most robust effects are seen in caregiver behavior and proximal child communication: parents increase responsiveness and reduce directive styles, mediating gains in social communication and early language. Child-level outcomes are more modest and heterogeneous. Some trials report improved communication; in a subset of samples, small reductions in early autism characteristics or lower probability of an autism diagnosis by 24–36 months are apparent, though these effects are inconsistent.

Cianfa et al. examine consequences of diagnostic timing in an Italian preschool cohort, finding that lower cognitive level and earlier recognition of developmental atypicalities predict earlier diagnosis, while lower adaptive social functioning, greater emotional and behavioral difficulties, and higher maternal stress characterize children diagnosed later. Their data suggest a “double disadvantage”: children with subtler profiles may be recognized after early intervention windows have passed, while their families carry a higher stress burden.

Together, these studies sharpen the ethical and practical tensions around intervening on likelihood rather than established condition. On one side, randomized evidence suggests earlier action can be efficacious: the iBASIS-VIPP preemptive intervention produced a sustained reduction in autism severity with lower diagnostic odds at age 3 (1), and Guthrie and colleagues demonstrated that even a narrow window of 18 versus 27 months meaningfully affects language and social communication gains (2). Delaying support until autism characteristics are fully consolidated risks narrowing developmental opportunities for at least some children. On the other side, while autistic development often reflects enduring neurodevelopmental differences, early signs in infancy can follow heterogeneous trajectories, with some children no longer meeting criteria over time. Accordingly, a neurodiversity-informed perspective cautions against framing preemptive programs as attempts to ‘eliminate’ autism, and instead emphasizes supporting each child’s communication, participation, and wellbeing across development (3, 4).. Autistic adults and their families consistently identify quality of life, functional communication, relational wellbeing, and family support as central outcomes—priorities contrasting significantly with changes in autistic characteristics or diagnostic status (5, 6). Integrating these perspectives requires identifying ways to promote socially-valid outcomes using practices acceptable to community members.

A constructive path through these tensions is to conceptualize early programs as development-enhancing, family-centered, and transdiagnostic, targeting functional domains such as social engagement, motor exploration, communication, and self-regulation rather than normalizing autistic traits (7). Several priorities follow for research and practice. Early identification should move beyond trait checklists to combine parent concerns, structured observation of social communication and motor skills, and, where feasible, biomarkers that help stratify likelihood and refine intervention timing. Service pathways should evolve toward tiered models offering low-intensity, relationship-based supports in primary care to all infants showing prodromal signs, with the option to step up as needed. Future trials should expand their outcome frameworks to include parental mental health, caregiver self-efficacy, parent-child relational quality, family quality of life, and child wellbeing alongside developmental measures, as these outcomes may carry equal or greater weight for autistic individuals and their families (6). The heterogeneity documented across this Research Topic confirms that there are multiple prodromal routes into later autistic and non-autistic outcomes, and that no single framework will serve all children equally.

The contributions in this Research Topic make it clear that we already have the tools to identify and support many infants with autism prodromes in their first years of life; the challenge now is to build systems willing, equipped, and mandated to act on this knowledge—while holding together the developmental efficacy and the relational, community-endorsed goals that define meaningful early support.
